# Paediatric Preference Prediction: the Future of Decision-Making for Children?

**DOI:** 10.1007/s12152-026-09669-x

**Published:** 2026-09-09

**Authors:** Dominic James Clifford Wilkinson, Julian Savulescu, Brian Earp

**Affiliations:** 1 Uehiro Oxford Institute, University of Oxford, Oxford, United Kingdom; 2 Newborn Care, John Radcliffe Hospital, Oxford, United Kingdom; 3 Centre for Biomedical Ethics, Yong Loo Lin School of Medicine, National University of Singapore, Singapore, Singapore

**Keywords:** Adolescent, Decision Making, Artificial Intelligence, Patient Preference, Decision Making, Shared, Treatment Refusal

## Abstract

Adolescents sometimes express firm views about high-stakes medical decisions that conflict with those of their caregivers or clinicians, raising difficult questions about how much weight their wishes should carry. A recurring concern is whether such decisions will later be regretted, although the normative significance of future potential regret remains hotly debated. Recent work in adult bioethics has proposed using artificial intelligence–based Personalized Patient Preference Predictors (P4s) to estimate what current treatment incapacitated adults would have chosen based on their known or inferred preferences when last autonomous. This paper explores whether, and with what ethical significance, a parallel ‘Paediatric Personalized Patient Preference Predictor’ (P5) could be applied to children and young people. We analyse how predicted *future* preferences might bear on questions of substituted judgment or proxy consent, best-interests assessments, resistance to treatment, and adolescents’ own deliberation. We distinguish different groups of paediatric patients based on current and future decision-making capacity, and identify both where a P5 might have greatest promise and where it is least defensible. We identify challenges for a P5 including that of self-reinforcing or self-negating preferences as well as of deciding which future preferences are relevant to current decisions. We argue that, in paediatrics, predicted future preferences cannot ground substituted judgment in the adult sense, but may nonetheless play a supportive role in best-interests reasoning and shared decision-making, subject to significant epistemic and ethical constraints.

## Introduction

Consider the following case

***Hannah’s choice***

*Hannah is 12 years old. She has a long history of illness, having been diagnosed with leukaemia aged 4. Following chemotherapy, Hannah developed cardiomyopathy as a side effect of treatment. Recently, Hannah has developed worsening heart failure and her doctors recommend that she be listed for a heart transplant. There are risks of this procedure, but without it, doctors feel that she is likely to die within months. However, Hannah does not wish to have the transplant, and wishes to be discharged home with palliative care. She is aware of the consequences of her decision. She explicitly says that she is not going to change her mind.*

Hannah’s choice is based on a UK case that received significant media attention in 2008 [1]. Some of the specific details of her case are uncommon. But there are many other situations in which adolescents have firm views about medical treatment that may differ from those of their caregivers. Some examples of these are listed in Box 1.

Box 1: Examples of controversial adolescent medical choices

**Table:** 

A 14-year-old identifies as a different gender from their birth sex, wishes to commence puberty blockers
A 15-year-old wishes to have cosmetic surgery because they are deeply concerned about their physical appearance
A 16-year-old Jehovah’s Witness with sickle cell anaemia does not wish to receive a blood transfusion

One of the key challenges in cases such as these is how to determine the weight that should be given to the young person’s wishes. Although the child or adolescent is sometimes adamant in their view about receiving or not receiving medical treatment, there may be uncertainty about whether it is the right thing to do as they request, or whether instead it would be better to act against their expressed wishes.

A common concern is that the young person may be making decisions that they will later regret. The significance of this is contested. Some authors argue that potential future regret is a normatively significant factor that should influence whether, say, an adolescent’s preferred choice is followed [2–4].^1^ Others argue, by contrast, that such regret should not be a decisive factor. They argue that what matters most from an ethical perspective is that a decision, at the time it is made, is sufficiently well-informed and well-considered [6]. According to this perspective, people, including adolescents, “have a right to be wrong” (or at least, a right to change their minds with time and perspective), without this invalidating their original decision [6]. We return later to this important point.

Our aim here is not to resolve disagreements about the precise normative weight that should be placed on the possibility or likelihood of future regret when deciding whether to honour a child or adolescent’s current treatment preference. Nevertheless, we suggest that if it could be known with a high degree of certainty that a young person would in the future continue to endorse, or at least not seriously regret their current decision, this would count as a prima facie reason in favour of honouring that choice. Conversely, and by the same logic, if it were known that they would be *highly likely* to change their mind or to regret their current decision, that would provide a prima facie reason to oppose their current request (or refusal). In the real case of Hannah Jones, she did later accept the transplant, and have the surgery. At age 18, she was quoted as saying “I'm glad I changed my mind at the time I did, otherwise I wouldn't be here today" [7].

Of course, a major challenge to including future preferences in decision-making for young people is that it can be very hard if not impossible to know with any great certainty what those preferences would be. But what if we could increase our level of warranted certainty about a young person’s future preferences?

For adults, some recent publications have explored the possibility of using artificial intelligence (AI) to predict the *current* likely preferences of an incapacitated patient, based—in part—on information about the preferences and values they had when they were last autonomous [8–11]. According to this view, so-called Personalised Patient Preference Predictors (P4) could play a key role in supporting proxy decision-makers in making substituted judgments. In this paper, we will extend that work to a paediatric population. If it were possible and sufficiently accurate, what would be the ethical significance of a *Paediatric* Personalised Patient Preference Predictor (P5)? (The question of how accuracy would be assessed or validated is a challenging issue to which we return later in our discussion.)

First, we outline some existing widely accepted ethical principles for paediatric (particularly adolescent) decision-making. (There are differences between jurisdictions in how these ethical principles are reflected in legal frameworks. Our aim here is to explore the ethical implications of paediatric preference prediction. A legal analysis of preference prediction is beyond the scope of this paper.) Next, we describe the ethical arguments in favour of patient preference prediction in adults lacking capacity. We then describe a theoretical extension of this to children and young people, identifying some of the ethical differences between adult and paediatric preference prediction, and the epistemic and ethical challenges in this population. This includes the challenge of deciding which future preferences are normatively relevant and why. We conclude with some thoughts about the ethical implications of preference prediction for cases like those listed above.

## Paediatric Decision-Making

For the purposes of simplicity, children and young people (henceforth CYP) can be divided into three groups based on their ability to make decisions (Box 2).

Box 2 Three groups of paediatric patients.

**Table:** 

CYP who have capacity to decide
CYP who have some ability to make decisions, but lack full capacity
CYP who have limited or no ability to make decisions

The youngest group of CYP (and some CYP who are older but have severe cognitive disability) have no capacity to make decisions themselves. The usual ethical principle that is applied in such cases is that medical decisions should be made in their *best interests* (ie what would best promote their overall welfare and health and avoid harms) [12]. So, if there were decisions about treatments that needed to be made for such CYP—for example relating to transplantation, hormone therapy, etc.—those would be based on a judgement about what would be best overall for them.

At the other end of the spectrum, CYP who have reached a threshold age or associated stage of maturity are assumed to be able to make at least some types of decisions in the same way as most adults.^2^ Accordingly, while other ethical principles may still play a role, the dominant ethical principle that is applied in such cases—at least in Western countries—is respect for patient *autonomy*.^3^ The patient’s wishes about treatment are considered to be crucial, and their decisions are generally respected even if others may regard them as unwise. If, for example, such a young person were to decline consent for a heart transplant, it would ordinarily be regarded as ethically impermissible (and potentially assault and battery) to proceed, no matter how beneficial the treatment were judged to be.^4^

In between these extremes, the majority of CYP have at least some ability to make decisions. It is generally accepted that the CYP’s wishes and opinions are respect-worthy, and that they should be involved in decisions about treatment to the degree that they are able [15]. However, it is also widely accepted that sometimes the CYP’s wishes should be overridden. For the good of the CYP, perhaps they *should* have medical treatment even when they do not, or strongly profess not to, want it. Sometimes, even though the young person clearly wishes for something to happen or not to happen, those caring for the CYP should not accede to that wish.

As we will argue shortly, this second group of CYP are those for whom a P5 might have most value. First, however, we set out the factors that might influence when the wishes of a CYP in this middle group should be overridden.

### Capacity

The first factor relates to the ability (“capacity”) of the young person to appreciate and understand the decision they are making, its positive and negative consequences over the short and long-term, and to weigh those up in reaching a decision.^5^ Since this ability exists on a spectrum, in theory the wishes of the CYP could have a variable amount of weight. In practice, the judgment that a CYP has capacity with respect to a given type of decision is usually applied in a binary fashion. If the young person has sufficient maturity and understanding, they are treated as a mature minor, meaning that they are granted the right to make even weighty medical decisions as if they were an adult. If they do not have this level of understanding/maturity, decisions are based instead on best interests.

Capacity requires explicit assessment. It is also usually understood to be decision-specific (ie the young person may be judged capable of making some decisions but not others) [16].

### Complexity and Consequences of Choice

One element of a decision that may influence whether a young person is judged capable of making it relates to its complexity. It is cognitively more demanding to make decisions that are affected by considerable uncertainty, have a range of effects (both good and bad), and where the effects are unfamiliar and/or potentially long-lasting.

A related factor concerns the seriousness of the consequences. Decisions that involve particularly high stakes potentially require much more careful deliberation and weighing up. They require a high-level appreciation of risk as well as sometimes reflection on difficult questions (eg on mortality). Furthermore, if a young person is making a decision that would potentially be seriously harmful, there is a stronger best-interests-based reason to overrule or disallow the risky choice.

An additional element is the potential for a decision to be irreversible. There may be greater concern about allowing young people to make decisions that are permanent, compared to ones that they could in the future reverse if they change their mind.

An example of a decision that is irreversible and has serious consequences is refusal of life-sustaining treatment (eg a transplant, or a blood transfusion). For the reasons that we have outlined, courts have sometimes chosen to override the refusal of CYP (even in cases where the young person is deemed to have capacity).^6^

### Resistance and the Consequences of Overriding Choice

Lastly, it is important to consider what would be involved in overruling a particular young person’s decision in terms of both their physical and mental wellbeing. For example, how are they expected to respond to such a decision; will they resist or accept a determination that is contrary to their expressed wishes; if they are distressed by it, how serious or harmful will the distress itself be, and how long will it last?

For example, in some cases, even if a treatment would in theory be in a young person’s best interests, it might not be worth pursuing if providing it would require repeated physical or chemical restraint. Conversely, it would be easier to justify imposing a treatment despite opposition if it could be provided quickly and simply without ongoing negative effects, or if were known that the young person would reluctantly accept the unwanted treatment.

Thus far, we have not mentioned one crucial element for paediatric decision-making: the views and wishes of parents and/or legal caregivers. There are different theoretical accounts of parents’ role in paediatric decision-making, and a full exploration of these is beyond the scope of this paper [17]. But one generally agreed principle (common to most accounts) is that while parents play an important role in making decisions for children who lack the capacity to make decisions by themselves, that role should be guided by (or limited by) the CYP’s best interests. Parents are not usually permitted to make decisions that would expectably cause serious harm to a young person [18].

With that background, we will now move on to thinking about preference prediction.

## Preference Prediction in Adults

Decision making in most adults is ethically more straightforward than in children. For adults who have capacity, the key ethical principle (as in older adolescents with capacity) is to respect patient autonomy and be guided by the wishes of the patient.

When an adult patient loses decision-making capacity because of acute physical illness or injury, mental illness or dementia, choices about their medical care are ordinarily made by a surrogate, for example a next-of-kin. In many parts of the world, the surrogate is not free to make any decision they personally prefer, nor even to do what they think would be best. Rather, the guiding ethical principle is that of *substituted judgment* [19].^7^

Substituted Judgement conceptually involves two elements. The first is to ask what we could call the ‘Counterfactual question’ –to identify what the patient would have decided if they were in the current circumstances and had the capacity to decide [20]. The second is a normative claim: the ethical choice is to make a decision that corresponds to the counterfactual determination. Where there is a nominated surrogate, this person is expected to make the decision that the patient would have made were they able to do so. Substituted judgement arguably shows respect for the patient by virtue of their former autonomy [9].

Empirical evidence, however, provides a challenge for substituted judgement. A systematic review found that in one third or more of cases surrogates incorrectly identified patient’s end of life preferences [21]. In another study, surrogates correctly identified the patient’s wishes about participating in research in only a little over a half of cases [22]. It is questionable whether substituted judgement meaningfully respects the patient’s autonomy if it has such poor correspondence with what the patient would (to the best of their knowledge) have wanted. Moreover, the weight of making consequential decisions, often with limited information, can be a source of considerable anxiety and moral distress for family members [23].

In response to these difficulties, Rid and Wendler proposed what they called a “Patient Preference Predictor” (P3), a statistical tool designed to generate evidence-based estimates of a patient’s likely choices [24]. They suggested that the P3 would draw on large-scale survey data linking demographic factors to treatment preferences, thereby offering probabilistic predictions to guide surrogates and clinicians. For example, if a sixty-five-year-old woman with late-stage cancer were facing a decision about whether to be admitted to intensive care with a chest infection, the P3 could predict her likely preference by comparing her demographic profile with the treatment choices reported by thousands of demographically similar individuals in prior surveys. This information could guide decisions and potentially relieve some of the burden on surrogates. Yet, reliance on population-level correlations has been criticised for neglecting the patient’s individual values and commitments [25]. On this view, respect for autonomy on a substituted judgment standard does not require that surrogates should merely reach “the right answer” about what the patient would hypothetically choose, but that they should decide based on the reasons *that specific patient* would rely on or endorse if they had capacity [26].

To address this concern, several authors recently proposed a “Personalized Patient Preference Predictor” (P4). Unlike the P3, which relies solely on group-level associations, the P4 would incorporate data specific to the individual patient—such as prior statements, writings, responses to structured interviews, or other personal information—potentially analysed using advanced computational methods such as fine-tuned large language models (LLMs). By grounding predictions in features more closely tied to the patient’s own values and life-history, a P4 may better respect them by virtue of their former autonomy and may also yield more accurate predictions of what the patient would have chosen [9].

Building on the P4 proposal, some of the same authors have since introduced the concept of a “digital life model” (DLM). A DLM extends personalised preference prediction from (a) helping with current decisions for an incapacitated patient based on information about their *previous* preferences and values, to (b) future-oriented self-reflection by competent adults, aiming to anticipate how they might later evaluate a current decision.^8^ Conceptually, a DLM is an advanced simulation that combines a personalised model of an individual’s known values, preferences, and commitments with a representation of the social and decision-making context in which those preferences may evolve [27]. Whereas P3 and P4 aimed to estimate what a patient would choose in present circumstances, DLMs aim to simulate how a person’s evaluative outlook might change across different hypothetical futures.

There remain both ethical and empirical questions about the use of a personalised preference predictor in adults (whether a P3, P4, or DLM). Those include, for example, the legal and ethical status of predicted preferences, their acceptability (for patients, families and professionals), and the accuracy of different forms of predictor.^9^ A number of those questions would apply similarly if analogous technology were to be deployed in paediatrics, particularly questions around the epistemology of assessing predictions for “accuracy” and how this could be validated (see Box 3 for details). In the following section, however, we wish to focus on the *distinctive* ethical questions raised by the prospect of personalised preference prediction in CYP. We will return to the epistemological challenges.

Box 3. Building and validating a Digital Life Model (DLM): Key epistemic challenges. Adapted with permission from Ong et al. [27]

**Table:** 

How could a digital life model (DLM) be constructed and validated? A central challenge for existing predictive preference proposals—such as the original P3 and its successor, the P4—is that they rely on individuals’ responses to hypothetical questions about what they would want under imagined conditions. In the P3 proposal, for example, population surveys link such hypothetical preferences to demographic characteristics, which are then used to predict what an incapacitated patient would prefer in a given situation. The P4 approach is more personalised, drawing on evidence about the known or likely preferences of the individual patient rather than relying solely on group-level correlations.
In both cases, critics have argued that people often cannot reliably anticipate how they will feel or decide until they are actually in the relevant circumstances. Their preferences are not necessarily preformed and static, but may often evolve over time in response to situations and stimuli. This concern motivates the DLM proposal (which presents a possible way of modelling how one’s preferences might indeed change over time under different conditions), but it also raises a validation problem: if people’s forecasts of their own future preferences are unreliable, then using those forecasts to train or validate a model risks circularity.
DLMs are intended to address this difficulty by combining personalised simulation with population-level grounding. Rather than extrapolating only from an individual’s past preferences, as in the P4 proposal, a DLM would be fine-tuned to a person’s current values, personality traits, and socio-relational commitments, and then used to simulate how their evaluative outlook might change across systematically varied future scenarios. Crucially, these simulations would be informed by empirical evidence about how people with similar characteristics have actually responded in comparable situations, in relation to how they anticipated that they would respond beforehand. (Determining what counts as sufficiently “similar” in terms of characteristics or “comparable” in terms of situations is important, though challenging).
In this way, DLMs neither simply reproduce an individual’s own speculative guesses nor rely exclusively on broad population averages. Instead, they aim to generate context-sensitive projections by integrating personalised modelling with observed regularities from comparable cases. Although such models cannot fully overcome the epistemic limits inherent in predicting future preferences (for example, the degree to which value-formation may be part of a highly specific physician-patient encounter), imperfect predictions may still be ethically and practically valuable, provided they are appropriately contextualised and not treated as authoritative.
Validation remains challenging given the counterfactual nature of the task. One plausible strategy would be to test DLMs in lower-stakes settings—such as predicting reactions to exam results, job outcomes, or minor health information—before considering their use in higher-stakes decisions. Demonstrated success in these domains could provide some evidence of applicability, without claiming definitive accuracy.

## Preference Prediction in Children and Young People

The extension of the P4 model to CYP would be a “*Paediatric Personalised Patient Preference Predictor” (P5).* We should start by evaluating the potential ethical uses of such a tool.

### Why Preference Prediction?

It is fairly self-evident that the current preferences of a young person are relevant to decision-making. But why would we wish to use a P5 to identify their predicted *future* preferences? In Box 4 and below, we set out different theoretical ways in which such future preferences could be relevant.^10^

Box 4: The relevance of future preferences for a Child or Young Person

**Table:** 

1. Informing substituted judgement
Which course of action would align with future judgement/autonomy
2. Informing Best interests
Which course of action would actually be best for them (as informed by self-knowledge)
Which course of action would best promote the patient’s values (value weighting)
3. Predicting resistance or non-resistance
4. Informing the child or young person about their likely future preferences, allowing further reflection

#### Substituted Judgement

One way in which these preferences could be relevant is through informing ‘substituted judgement’. As noted above, this involves determining what the individual patient would have decided or preferred if she or he were able to do so [29]. There are challenges in determining what the former adult patient with capacity would have chosen, not to mention unresolved philosophical questions about what “hypothetical” consent or endorsement even means or amounts to (ie, what it reflects and its normative force) [30]. For CYP, substituted judgement is even more challenging. We will return to some of the reasons behind this shortly, but one important point to note is that when we think about predicted preferences, there are two different questions that we might ask:

The Counterfactual question: What decision would the future adult with capacity make if they were somehow present in the current moment (ie if they could come back in time, visiting the present from the future)?

Future Endorsement question: What decision would the future-self endorse (ie be glad had been made) or regret?

Most information about future values and preferences will be relevant to the endorsement question. But that answer may or may not be the same as the counterfactual question. This might be, for example, because the person has significantly changed or even been transformed by the very experience of having lost capacity and been treated [31]. We will return to some of these challenges (including that of potential self-reinforcing and self-negating preferences) in a later section.

In theory at least, substituted judgement in paediatrics might help identify which course of action would respect the patient’s future autonomy. It was clear in Hannah’s case that her older-self thought that it was a good idea to have a transplant and presumably would have chosen this option for her younger self. (Of course, in the counterfactual scenario in which the transplant were refused, Hannah might not have had an older-self.) If this had been able to be predicted, perhaps that substituted judgement might have been used to justify surgery?

#### Best Interests

A person’s views about treatment are potentially relevant for reasons of *beneficence* (as well as for reasons of autonomy). Thus, a P5 might help to identify which option would best promote the patient’s *interests*. Self-knowledge is not infallible, but potentially the future person may know the facts of their life, their personal circumstances, and how they respond to adversity better than anyone else [32]. The predicted views of the patient’s future self also offer a plausible view about how to evaluate the likely outcomes in the light of what matters to them – ie their values.

#### Resistance

We alluded earlier to the impact of *resistance or non-resistance*. If a P5 were to predict that a patient will persist in mounting strong opposition to treatment (perhaps physically resisting any attempts to treat them), or conversely that they will ultimately accept the treatment if they are required to, that could be relevant to determining whether it is a good idea to overrule them.

#### Support for Current CYP Decision-Making

Finally, future preferences might also be relevant to the child or young person and their current wishes. In theory, receiving reliable information that you will change your mind in the future, ought to lead someone to revise their point of view – or at least to reduce their confidence in their current opinion. For example, if an adolescent were told that it is likely that in the future they would no longer hold their current strong view against receiving a blood transfusion, that might make them reconsider their current opposition. (Whether this advice will be believed or heeded is another question.)

### Different Forms of Preference Prediction in CYP

Following our earlier suggestion that paediatric patients can be roughly grouped (based on their capacity), we can divide preference prediction in a way that is similar, albeit slightly different (Box 5).

Box 5 – Preference prediction and groups of paediatric patients based on current and future capacity.^11^

**Table:** 

1. Prediction in CYP with current capacity
2. Prediction in CYP with reduced or no capacity, but future capacity
3. Prediction in CYP with reduced or no capacity both at present and in the future

#### Group 1: CYP with Current Capacity

Addressing these in turn, the first possibility would be to use a P5 in the case of older adolescents who are judged to currently have decision-making capacity. For example, in Hannah’s case, which we opened with, she was judged by a number of those who talked with her to have sufficient maturity and understanding to make the decision. But if she were predicted to change her mind, this might have been used as a reason *not* to allow her to refuse transplantation, or, at least, to try to persuade her that her current conviction might not be as stable and long-lasting as it now seems.

The first thing to say about this possibility is that it is potentially misconceived. For adult patients with capacity, respect for autonomy implies respecting decisions (at least, treatment refusals) even if the health professional disagrees with it, indeed even if there is good reason to think that the patient is making a mistake. To put it most forcefully, even if we knew with 100% certainty that an adult patient would later change their mind and regret their current decision, their choice should be respected. (Call this the ‘right to be wrong’ view). There are some authors who have questioned this conclusion for adults [31]. Nevertheless, the standard ethical and legal approach endorses the idea of a ‘right to be wrong’. *If* we take this view of decisions for adults, *and* a CYP is thought to have adult-level decision-making capacity, then, so long as the analysis is confined solely to the question of decision-making capacity, and any other differences between adolescents and adults are set aside, it follows that preference prediction should not be used to overrule their decisions. Even if an appropriately validated P5 were to indicate with a high degree of justified certainty that future Hannah would wish for the transplant, on this view we should respect her current refusal of treatment anyway.

A different view is also conceivable. Autonomy might be given lesser weight in adolescents compared to adults. This would potentially involve acting in the best interests of young people when autonomy and beneficence conflict. A P5 might provide support for a determination that it would be in the best interests of an adolescent like Hannah to receive treatment against their wishes. It would represent a form of paternalism, but might be defensible because of a duty to protect CYP from self-harming choices. If we took such a view, this would deny that adolescents have a ‘right to be wrong’ – at least in terms of some high stakes choices. It would align with some legal decisions in which adolescents have not been permitted to refuse a blood transfusion even though they appear to have decision-making capacity.^12^

A less controversial possibility would be, as noted in the previous section, for a P5 to inform the young person about their likely future wishes, and therefore help to support them in making a fully autonomous decision. There is a deep philosophical question about whether preferences which are not based on one’s values are autonomous [33] and this extends to whether present preferences not based on one’s future values are autonomous. Another deep philosophical issue is how to respect and treat conflicting preferences and values over time. Parfit famously imagined an idealist young Russian Nobleman who wants to devote his money and resources now to the Revolution but in the future will value material wealth and comfort [34]. Which values ought to guide his actions? There are several possibilities for how this conflict of values could be resolved in CYP with capacity. Perhaps the values which best promote their well-being, objectively conceived, should be respected. Perhaps the values which keep most options open for them (freedom) should be respected. Or perhaps the values which are strongest, or most deeply held ought to be given priority. We cannot settle these issues here save to say that there are various reasons why future values ought to be presented to the present person, insofar as these can reliably predicted.

For the purposes of this paper, we will not try to settle the question of whether or how a P5 should be used in this group of CYP (those with capacity). To a large degree, the ethical use in that group will overlap with the use of a DLM (see Box 3) in adults. We will focus for most of the rest of the paper, on another group of CYP.

#### Group 2: CYP with Reduced or No Capacity, but Future Capacity

The largest group of patients in paediatrics are those who currently lack the ability to make decisions for themselves, but who will have this capacity in the future, if all goes well. It is also for this group of patients that there appears to be the strongest case for P5.

The ethical case here is less controversial than in Group 1 because, by definition, this group of patients has *reduced* decision-making capacity. It is therefore less problematic to overrule their current wishes on the basis of predicted preferences. This is also a group of patients where (in theory at least), it would be possible to obtain personalised data to inform prediction of their future preferences. There are some epistemic challenges (we will return to these shortly, as promised), but it appears most attractive to attempt preference prediction in this group of patients.

It is worth noting that patients in ‘Group 2’ range from young children to adolescents and correspondingly have varying degrees of ability to make decisions. This will be relevant to the methods of preference prediction. It will also be relevant to the accuracy of preference prediction.

#### Group 3: CYP with Reduced or No Capacity Both at Present and in the Future

Finally, some young people lack current decision-making capacity, but also will never be in the position to have capacity. That includes those with intellectual impairment of varying degrees, as well as those with life-limiting illnesses. As in ‘Group 2’, it includes some who have no current ability to express preferences, and others who may have views and values, albeit they lack full capacity.

Any attempt to predict preferences in this population would involve significant extrapolation. For example, a P5 could take children and young people of a particular age and background (perhaps also with similar current views) and identify the future preferences of those who do not have a life limiting illness or cognitive impairment. However, unlike other groups of patients (and notwithstanding some further problems discussed below), there will never be any way of verifying the accuracy of this on an individual level. It would not be possible, for example, to ask in the future any of these patients whether they endorse a particular decision made in the past. Nor will it be possible (by definition) to find any adult patients with capacity who were once in ‘Group 3’ to ask them about their views on treatment. Furthermore, we may be sceptical about the assumed comparability of preference development in those with and without impairment/life-limiting illness. It is highly likely that having a significant impairment in cognition, or facing the real prospect of your death prior to adulthood would have a significant effect on the things that a CYP values and on the decisions that they reach. If that is correct, then we should not expect that the views of CYP who do not have an impairment, and/or who survive to adulthood to be the same.

One possibility for such patients would be less ambitious. Prediction could be attempted – not of their future preferences with capacity, but of their future preferences without capacity. Imagine, for example, a young person with moderate cognitive impairment who does not wish to have a surgical procedure. Predictions could be made to identify whether at a future time point (when they still have cognitive impairment), they would be likely to regret not having had the operation (ie answering the Endorsement question). There are interesting further questions about the significance of such preferences. At the very least, we should observe that they are different in nature from future autonomous preferences.

Given the impossibility of verifying the accuracy of future predicted preferences in this group (at least, vis-à-vis an autonomous self-endorsement standard), and that conceptually, preference prediction could also not be justified on the basis of future autonomy (because the future self will never be strictly autonomous), we suggest that preference prediction in this group of patients is likely to be inappropriate. The ethical basis for decisions should remain the patient’s best interests. This can (and should where possible) include giving some weight to the patient’s current preferences and values. But their future preferences, predicted by a P5 or some other method, would have no formal role.

### The Methods of Preference Prediction in CYP

The central approach to prediction preferences in a P5 would in theory be similar to those described in adults. It would involve some combination of data from large population data sets (similar to those described for a P3), in conjunction with a variable amount of personalised data (as described for a P4). As described in Earp et al., there is a variety of different forms of personal data that might be included [10]. These might include personal writing or online activity from which relevant preferences and values can be reliably inferred. It could include previously expressed preferences and decisions.

One point to note is that depending on the circumstances (eg the age of the CYP, as well as choices about which data to include or exclude), some paediatric patients might have more personal data such that predictions would be more personalised (akin to an adult P4), while others would have less data, such that predictions would have to be inferred primarily from population data (as in an adult P3). It might be, for example, that preference prediction for very young children is able to include only the latter type of information.^13^

However, one potential difference from an adult P4 is the possibility for the CYP to provide contemporaneous information about their views and preferences. For example, an adolescent could answer a set of questions about their perspectives on treatment, how they would evaluate the risks and benefits of different options. Alternatively, they could engage directly with an LLM that aims to assess the nature and strength of preferences. In their description of the adult P4, Earp et al. suggest that one source of personalised values might come from patients willing to participate in “specially designed, value-eliciting discrete choice experiments … in which they would need to decide between options in a sequence of tradeoffs pitting various decision-relevant factors against one another” [10]. These could “potentially be ‘gamified’ and delivered by way of a downloadable mobile app, [or] an appropriately secured computer interface” [10]. By the nature of the situations in which a P4 might be used (i.e., largely in cases of sudden complete loss of capacity), adult patients are unlikely to be able to provide any *current* input of this sort, and perhaps only a small proportion will have done so in the past. But the adolescent patient might be able to do this, and thus enable a stronger, more direct form of personalised input for predictions.

## Challenges and Counterarguments

We have set out some of the ways that a P5 might be useful, the group of patients in whom it has most promise (“Group 2”: those paediatric patients with reduced capacity at present, but future capacity), and the methods (broadly speaking) that could be used for predictions. However, it is important to acknowledge that paediatric predictions face some unique challenges.

### Epistemic Problems for the P5

First, the evidence required to inform decisions (and to assess the accuracy of predictions) will be more difficult to collect because of *time*. For adult patients, it would be possible to ask current patients who have capacity their views about treatment. These could be compared with the outputs of P3, or P4, to assess the accuracy of predictions. But for CYP, this exercise would necessarily require collecting current views of patients, and then waiting for the passage of time to assess their views when they have capacity. Predictions for younger patients will require a longer period of study (research will be harder to perform), but might also be associated with greater uncertainty because of the potential for other factors (for example other life events) to influence value and preference formation or change.

Second, as noted earlier, evidence collected later in life will most often tell us whether the later person is glad that a particular decision was made, or would prefer that a different decision had been made. But that is not necessarily the same as the decision that *they would have made*. Later endorsement/regret might be useful to know, but is a conceptually different answer.

There is a related, deeper challenge. This is the problem that in a number of cases, treatment decisions may themselves influence or bias the evolution of later preferences (or even of later selves). Choice-contingent preferences might be of two kinds: self-reinforcing preferences or self-negating preferences. Consider first:

Self-reinforcing preferences:

If make choice A: more likely to have later preference for choice A

If make choice B: more likely to have later preference for choice B

Reinforcing preferences could arise where an individual incorporates a particular decision into their self-conception. This might be especially likely for significant decisions made during adolescence, a period when identity is often in flux and perhaps most capable of being swayed. One theoretical example where preferences might have this character, would be where an adolescent chooses to take puberty blockers to prevent the development of secondary sexual characteristics. It has been claimed, albeit controversially, that adolescents who embark on such treatment are more likely to later choose to take cross sex hormones, while those who have gender dysphoria but do not take puberty blockers are less likely later to choose to take hormone therapy [35].^14^ If that claim is correct, one potential mechanism could be through the influence of having made that decision on subsequent gender identity. An alternative perspective on this evidence might be that those adolescents who commence puberty blocking medication have been correctly predicted to (in the future) persist in their gender identification (ie a selection effect). For this paper, we do not take a side on which of these explanations is correct – only to point out that the possibility of impact of decisions on later preferences makes the interpretation of data much more challenging.

However, choices could also influence later preferences in a different way:

Self-negating preferences:

If make choice A: more likely to have later preference for choice B

If make choice B: more likely to have later preference for choice A

In situations where patients face difficult choices, there is the potential for decisional regret [37]. Indeed in some cases, all available options may have significant negatives, and whatever decision is made, patients may experience a form of “buyer’s remorse.” They may wish that they had made a different choice.

The process of decision-making may also influence future preferences. For example, in some cases coercion or a forced decision might entrench opposition. In Hannah’s case, the fact that she was permitted to decline treatment may have given her the psychological space to later change her mind and accept treatment. It is possible that if treatment had been applied against her will, she may have remained opposed and later viewed the decision negatively. In an additional complication, it is possible that preference prediction itself might influence later views of the patient (as a form of self-fulfilling or self-negating prophecy) [38].

One significant challenge for the use of P5 in CYP is that their identities and values are not fully shaped but are in flux. The preferences they have now and in the future may or may not cohere with their future (relatively) settled values. Those values may be shaped by their choices now (as we have pointed out) or by a whole range of factors in and out of their control, including specific encounters with health professionals in the future. This problem is not unique to adolescents, nor will it always apply. Nevertheless, it creates a challenge for both making predictions and acting on them.

Lastly, and as alluded to previously, some decisions may, by their nature, prevent a future self from existing to have autonomous preferences about treatment. If, for example, Hannah had died at age 13 following her decision to decline a transplant and receive palliative care, we could have no information about whether she would have later endorsed or regretted this choice. In such cases, such evidence as we have about future preferences will be asymmetric – only decisions that lead to longer term survival (with capacity) will generate evidence about preferences.

Whether we are trying to answer the counterfactual question or the endorsement question, there may not be a simple or single answer. The best answer may be ‘it depends’. The epistemic problems do not mean that prediction is without value. However, its normative significance may change.

### Uncertainty

Predicted preferences are always going to be associated with some degree of uncertainty about what the individual would have wished. If it were necessary to know for certain what the individual would have wanted, then substituted judgement would never be acceptable except where a patient has made a formal advance directive. (Even where the patient has made such a directive, there can be some uncertainty about whether it is applicable, and/or whether the patient would have changed their mind since they wrote it.) In most jurisdictions absolute certainty is not thought to be necessary when making decisions for patients lacking capacity. It suffices that there is *enough* confidence in the patient’s wishes. However, there are two uncertainty-related challenges for the P5.

The first is that the epistemic challenges identified above mean that there will often be *greater* uncertainty for predicted preferences for CYP compared with adults. That potentially means that there will be a smaller proportion of cases where there is sufficient accuracy for decision-making.

The deeper problem is that one of the arguments relating to acceptable uncertainty in relation to the P4 in adults doesn’t work in the paediatric case. As noted above, existing decision-making for adult patients depends on surrogates and substituted judgement, but studies indicate that surrogates are not very accurate at correctly predicting the wishes of the patient. In that context, preference prediction that is *more accurate* than surrogates would be theoretically advantageous – even if it cannot be certain. For example, if a P4 were to have 80% accuracy for predictions, that may be worth using – assuming it is better than the default. But one problem for the P5 is that existing decisions for CYP do not currently rely on predicting the young person’s future wishes. Instead, decisions are based on the patient’s best interests. Would a prediction of the future CYP’s wishes (with 80% accuracy) be better than an assessment by parents or professionals of Hannah’s best interests? That is difficult to answer.

### Decision-Making Authority and the Timing of Preferences

For adults, the question of whether substituted judgement is more accurate than a best interests assessment does not arise. That is because the wishes and values of the formerly autonomous patient are thought to have precedence. Another way of stating this is that the previous self has decision-making authority over the later self without capacity.^15^ The justification is that the earlier self has a stake in their future: they hope that if they should lose the ability to make decisions, their (current) wishes will be respected. Assuming the person has not changed too much in the meantime [39], their earlier preferences persist and are highly relevant to decisions for the person who has lost capacity. For these reasons, it is plausible that the earlier self has a *right* to make decisions for themselves after they lose the ability to decide.

However, these claims do not apply in the same way for future preferences and past selves. When we are making decisions for CYP, their future self does not yet exist, nor do their future values and preferences. There are philosophical debates about whether, and if so in what form, future people can have rights [40]. However, even if we think that they can have some rights, it is hard to imagine that future selves can have the right to *decide* for their past selves—they cannot have decision-making *authority*. (As noted earlier, depending on the decision that is made, whether the future self exists, or *which* future self exists, may depend on the decision that is made. It would be incoherent to, for example, overrule the wishes of the current Hannah on the basis of the authority of a future Hannah who, if not for this very action, would never exist.)

This is relevant to the question of which future time point would count for the purposes of predictions. If we were using predictions in a way that is analogous to substituted judgement in adults, we might use the preferences of the closest future self with capacity. For example, this could be their preferences at (say) age 18. However, as noted, the future self lacks clear normative authority.

A different rationale might be that if, hypothetically, it were possible to defer decision-making, there would often be strong reason to do so. If decisions were able to be deferred, it is these young adult preferences that would then direct decisions. For example, some forms of cosmetic procedure (surgery, or tattoos) are not permitted under the age of 18 in many jurisdictions.^16^ However, once the young person reaches this age, assuming they have capacity, their wishes then become determinative. But if that follows, and a P5 were able to predict the preferences of the future young adult self, we might not need to wait until the young person reaches a threshold age, or attains capacity.

But deferring decisions may not be possible (as indeed was the case for Hannah). In that case, we have to decide whether to be guided by the wishes of the hypothetical future self. If the future self lacks authority to decide, we suggest that the most plausible alternative is that future preferences for CYP are relevant *not* as way of informing substituted judgement, but rather through their relevance for what would be best overall– ie for an assessment of best interests.^17^ However, in that case the question of which preferences to predict and use becomes even more challenging. It is conceivable that preferences in early adulthood are not necessarily stable, and that somewhat older preferences would be more predictive of the views of the adult self. On the other hand, if the aim were to minimize future regret, it would be important to know the views of the individual over time and how or whether those change, perhaps aiming for the most *diachronically stable* set of values. This sort of prediction would be even more difficult to make and substantiate.

If we were to use a P5, we would need to understand preferences over time. Such predictions could help us to identify whether a young person would be likely persist in their view (for example in holding a particular religious view about treatment, or in having a particular gender identity), or alternatively whether they would be likely to change their mind and regret a particular choice (eg to decline a transplant). As noted earlier, that could be relevant to the person’s best interests because of how their choice will interact with their future preferences, and values. Even setting aside uncertainty, these preferences would not settle the question about what would be in the person’s best interests. There may be cases where the future person is predicted to continue to hold a view that is factually mistaken and endorse a decision that is actually contrary to their overall interests (eg that most vaccines are overall harmful, or that non-scientifically based remedies are all that is required to treat a cancer). Even if we could be certain about future preferences, there is no guarantee that the future person will endorse or choose the best option for their younger self. In that case, their future view would be relevant, but other decisions may appropriately be reached.

The deeper philosophical problem is the need to decide which future preferences matter for the purposes of decision-making. A person may evaluate the same intervention differently at different stages of life, and plausible accounts may privilege duration, stability, later reflection, or the perspective taken near the end of life (when an individual reflects on their life as a whole). Prediction alone cannot adjudicate among these standards. Any use of a P5 must therefore be embedded within an independently justified account of which future preferences count, how conflicts among them should be resolved, and what role those preferences should play alongside the child’s present interests and views.

## Conclusions

In this paper, we have described a potential extension of an existing ethical proposal, to use data from a variety of sources to provide personalised preference prediction, to the case of paediatric patients. At the present time, the technology does not exist. Yet there is reason to think that it would be possible. We have described some of the theoretical benefits and limits of such an approach.

There are some groups of paediatric patients where a P5 would have at best a limited role.

For very young patients, preference prediction may be associated with too much uncertainty to be useful. Much may happen during childhood and adolescence that could influence the values they will hold as an adult. Limited personalisation would be possible because of the lack of information on values and preferences that are likely to endure.

For patients who will never have capacity (for example, those with life limiting illness or cognitive impairment), preferences would be hypothetical and it is not clear in this group what, if any, ethical role they should play.

A P5 would be of most use, we suspect, in adolescent patients with future potential capacity like Hannah, whose case we described at the start of this paper, or some of the other examples listed in Box 1. We have argued that the most important role of predicted preferences would be helping to identify the best interests of young people, through identifying decisions that they will likely endorse, or not regret, and that are predicted to align with their (diachronically relatively stable) values. There are some significant epistemic challenges in determining what the future young person would choose or endorse (including the problem of preferences that are likely to be influenced by treatment decisions or anyway change), but there may be at least some cases where it would be possible. There are also challenges in identifying a defensible account of the temporal authority of future preferences. This limits the plausibility of treating P5 as a straightforward analogue of substituted judgement.

We have noted, but not resolved, how a P5 might be used in older adolescents who appear to have capacity. Adults (as long as they have capacity) are ordinarily permitted to make self-harming choices – even where it is predictable that they will later change their mind and regret that decision. If we take a similar view of mature minors, then predicting future preferences would not provide grounds for overruling an adolescent with capacity. However, it would still be important to reveal these to the deciding adolescent and engage them in dialogue about their significance. This could provide support in a similar way to how a Digital Life Model has been proposed as a decision-support and reflection-stimulating tool in adults [27]. But as we alluded to earlier, a different response would also be defensible. If we gave lesser weight to autonomy in young people, a P5 might be useful in providing evidence that the young person would or would not change their mind with the passage of time, and therefore in some cases support overruling their decision in their best interests.

A key element of decision-making for children is the potential to affect, for good or bad, the adult who they will become. There is a well-founded desire to protect and promote the wellbeing of that future person, but it is not always easy to identify which option will secure that. Preferences derived from a P5 would not be determinative. But they might, nevertheless, be helpful for parents, health professionals and perhaps also the young person.

## Data Availability

No datasets were generated or analysed during the current study.

## Footnotes

1 See also more generally, not just about adolescents [5].

2 This age varies between jurisdictions, and in some places there is more than one age threshold. For example, in England and Wales, young people who have passed their 16th birthday are usually able to consent for treatment in the same way as adults (ie unless they lack capacity). Those who are 18 or above, are presumed to have capacity to consent, and may refuse any medical treatment.

3 In this paper, we use "autonomy" and “autonomous preferences” to refer to preferences that the person reflectively endorses as expressing their settled (ie diachronically reasonably stable) values and commitments, rather than a preference that is merely momentary, impulsive, or externally imposed. The Belmont Report (1979) referred to ‘respect for persons’ where persons need not only be understood in the technical sense of autonomous beings. In today’s bioethics literature, people often gloss respect for persons as respect for autonomy, but that is arguably a narrowing of the original [13].

4 For life saving treatment, courts have often over-ruled adolescents (judged to have capacity) in order to safeguard their best interests (eg Wheeler) [14]. The question of whether this is justified lies outside the scope of this paper.

5 Within bioethical literature, this ability is sometimes referred to as “capacity”, and sometimes as “competence”. These terms sometimes also refer to distinct legal concepts. For the purposes of this paper we use ‘capacity’ generically to refer to the ability to make decisions that, ethically speaking, generally ought to be honoured even if others regard them as unwise.

6 See note 4

7 In the UK, ‘substituted judgement’ is not part of the formal legal framework model for adults (instead, decisions are based on the adult’s best interests). However, evidence about what the patient would have decided if they had capacity has very substantial weight in best interests determinations.

8 In this respect, it is the closest existing analogue to a P5, although it does not address the distinctive ethical issues raised by paediatric decision-making, which are our focus here.

9 For a comprehensive analysis of some of these see [28]

10 Below, we argue that the some of these ways are more ethically significant than others.

11 Note that this is different from the earlier division. The former (Box 2) was based purely on current decision-making capacity. The division here includes a separate group who currently lack capacity *and will never have capacity*.

12 See note 4

13 One form of personalisation that might be possible in young children would be to include parental preferences. The idea would be that children of parents with certain world views/values are more likely to have a particular set of preferences in the future when they have capacity. The prediction model would not simply assume that the CYP will necessarily share the parents’ views about treatment in the future. It may be predictable that the CYP would *not* share those views. Instead, it would look at evidence on preference development, and seek to estimate the CYP's likely views in the future given a particular background etc.

14 Some studies reach the opposite conclusion: that prescription of puberty blockers is not associated with an increased later use of gender affirming hormones [36].

15 Theo Naylor has argued that the earlier self has decision-making authority to the degree that the previous self is psychologically continuous with the later self [39]. Such authority is weakened in cases where the previous preference is relevantly like a preference about a different individual.

16 Another example is assisted dying, which is restricted to those over a threshold age in some countries.

17 There is a further possibility – beyond the scope of this paper, that AI/large language models might be used to predict not preferences but wellbeing – ie the future level of happiness.
